# The ryanodine receptor stabilizer S107 ameliorates contractility of adult Rbm20 knockout rat cardiomyocytes

**DOI:** 10.14814/phy2.15011

**Published:** 2021-09-14

**Authors:** Wei Guo, Chaoqun Zhu, Zhiyong Yin, Yanghai Zhang, Chunyan Wang, Andrea Sanchez Walk, Ying‐Hsi Lin, Timothy A. McKinsey, Kathleen C. Woulfe, Jun Ren, Herbert G. Chew

**Affiliations:** ^1^ Department of Animal and Dairy Sciences University of Wisconsin‐Madison Madison Wisconsin USA; ^2^ Department of Animal Science University of Wyoming Laramie Wyoming USA; ^3^ Department of Biology Western Wyoming College Rock Springs Wyoming USA; ^4^ Division of Cardiology, and Consortium for Fibrosis Research & Translation Department of Medicine University of Colorado Anschutz Medical Campus Aurora Colorado USA; ^5^ School of Pharmacy University of Wyoming Laramie Wyoming USA; ^6^ Department of Pharmacology University of California Davis California 95616 USA; ^7^ Department of Cardiovascular Medicine Xijing Hospital Fourth Military Medical University 15 Changle West Road Xi'an Shanxi China

**Keywords:** alternative splicing, Ca^2+^ signaling, dilated cardiomyopathy, RBM20, S107

## Abstract

RNA binding motif 20 (RBM20) cardiomyopathy has been detected in approximately 3% of populations afflicted with dilated cardiomyopathy (DCM). It is well conceived that RBM20 cardiomyopathy is provoked by titin isoform switching in combination with resting Ca^2+^ leaking. In this study, we characterized the cardiac function in Rbm20 knockout (KO) rats at 3‐, 6‐, 9‐, and 12‐months of age and examined the effect of the ryanodine receptor stabilizer S107 on resting intracellular levels and cardiomyocyte contractile properties. Our results revealed that even though Rbm20 depletion promoted expression of larger titin isoform and reduced myocardial stiffness in young rats (3 months of age), the established DCM phenotype required more time to embellish. S107 restored elevated intracellular Ca^2+^ to normal levels and ameliorated cardiomyocyte contractile properties in isolated cardiomyocytes from 6‐month‐old Rbm20 KO rats. However, S107 failed to preserve cardiac homeostasis in Rbm20 KO rats at 12 months of age, unexpectedly, likely due to the existence of multiple pathogenic mechanisms. Taken together, our data suggest the therapeutic promises of S107 in the management of RBM20 cardiomyopathy.

## Graphical abstract

Loss of Rbm20 results in reduced myocardial stiffness at younger age and overt RBM20 cardiomyopathy at older age in rats. The RyR2 stabilizer S107 improves contractility properties by restoring the elevated resting intracellular Ca^2+^ level in Rbm20‐depleted cardiomyocytes. S107 could be a component of a therapeutic option for RBM20 cardiomyopathy.

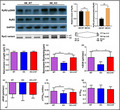

## 1 INTRODUCTION

Cardiomyopathies are among the most prevalent causes of premature death in developing and developed countries. RNA binding protein 20 (RBM20) cardiomyopathy is a recently identified unique form of cardiomyopathy afflicting approximately 3% of patients with dilated cardiomyopathy (DCM) (Brauch et al., 2009; Guo et al., 2012; Linke & Bücker, 2012; Refaat et al., 2012). RBM20 is a splicing factor that is predominately expressed in muscle tissues with the highest expression level in the heart (Guo et al., 2012, 2013; Li et al., 2012, 2013). RBM20 primarily regulates gene splicing of TTN, encoding a third abundant sarcomeric protein titin, which plays a determinant role in ventricular wall stiffness (Granzier & Labeit, 2002, 2004; Guo et al., 2012). Titin‐based ventricular wall stiffness can occur in two ways. One is fine‐tuned through post‐translational modifications (PTMs), particularly through the phosphorylation and dephosphorylation of titin's spring domains (Hamdani et al., 2017; Hidalgo & Granzier, 2013; Krysiak et al., 2018; Tharp et al., 2019). The other is regulated through switching titin isoform ratios. In the heart, titin has two isoforms known as N2BA and N2B isoform resulting from alternative splicing. The N2BA isoform is larger and more compliant since it contains a longer PEVK (proline (P), glutamate (E), valine (V), and lysine (K)) domain and more spring‐like Ig domains, while the N2B isoform is shorter and stiffer due to its short PEVK region and few spring‐like Ig domains (Guo et al., 2010; Guo & Sun, 2018; Tharp et al., 2020). In healthy human hearts, the ratio of compliant N2BA to stiffer N2B is typical about 30–70 (Makarenko et al., 2004). However, in human patients, the ratio was overtly elevated in ischemic cardiomyopathy (Neagoe et al., 2002), non‐ischemic DCM (Nagueh et al., 2004), and patients with heart failure with a reduced ejection fraction (HFrEF) (Borbély et al., 2009).

Rbm20 missense mutations identified in human patients with DCM and animal models result in expression of the larger N2BA titin isoform (Brauch et al., 2009; Ihara et al., 2020; Li et al., 2010; Refaat et al., 2012; Schneider et al., 2020), and deletion mutations with loss‐of‐function of RBM20 in animal models also lead to expression of the larger titin isoform and DCM in animal models (Guo et al., 2012; van den Hoogenhof et al., 2018; Khan et al., 2016). Therefore, it is speculated that the presence of the largest titin isoform caused by Rbm20 missense mutations in human patients and loss‐of‐function mutations in animal models serve as the major underlying mechanism en route to DCM. However, this notion was challenged by a mouse model developed by Dr. Henk Granzier's group in which deletion mutation with truncated RBM20 expression causes expression of the larger titin isoform but not DCM phenotype (Methawasin et al., 2014). These studies imply that titin isoform switching is not the only cause for DCM in RBM20 cardiomyopathy. Evidence from our group and others found that RBM20 also regulate alternative splicing of over 30 other genes in addition to TTN. These genes include those that encode regulators of Ca^2+^ handling, such as ryanodine receptor 2 (RyR2), Ca^2+^/calmodulin‐dependent kinase II‐delta (Camk2d), and triadin (Trdn) (Guo et al., 2012; Maatz et al., 2014). More data have noted onset of ventricular arrhythmias and increased basal intracellular Ca^2+^ release in RBM20 cardiomyopathy (Guo et al., 2018; van den Hoogenhof et al., 2018).

In this study, we further characterized the effect of RBM20 on the progression of heart failure with development in Rbm20 knockout (KO) rats. We evaluated age‐dependent changes in cardiac function and myocardial stiffness at multiple time points in WT and KO rats using echocardiography and pressure‐volume loop analysis (PV‐Loop), and quantified myofilament stiffness in 3‐month‐old rats compared to 12‐month‐old rats. Moreover, we assessed contractile properties and Ca^2+^ dynamics in isolated adult cardiomyocytes from 6‐month‐old rats. We then treated both in vitro isolated adult cardiomyocytes and Rbm20 KO rats with the ryanodine receptor stabilizer S107 to test whether S107 can inhibit the increased basal Ca^2+^ release in vitro and thus improve cardiac function caused by Rbm20 deletion mutation in vivo.

## 2 MATERIALS AND METHODS

### 2.1 Experimental animals and in vivo treatment

This study was performed with wild type (*Rbm20* ^+/+^, WT) and homozygous knockout (*Rbm20* ^−/−^, KO) rats. The Rbm20 KO rats were derived from a spontaneous mutant (Greaser et al., 2008; Guo et al., 2012). Rats used in the current work were crosses of Sprague‐Dawley (SD) X Brown Norway (BN) (All strains were originally obtained from Harlan Sprague Dawley). Animals were maintained on standard rodent chow. This study was carried out in strict accordance with the recommendations in the Guide for the Care and Use of Laboratory Animals of the National Institutes of Health. The procedure was approved by the Institutional Animal Use and Care Committee of the University of Wyoming. Male WT rats and male KO rats, age 3–12 months, were used in this study. For S107 treatment, 12‐month‐old male WT rats (8 rats per group) were placed on normal drinking water (control group) and 12‐month‐old male KO rats (9 rats per group) were put on drinking water with 20 mg/kg/day S107 (Shan, Betzenhauser, et al., 2010) (experimental group). Male KO rats were treated with S107 for 2 weeks.

### 2.2 Echocardiography

For in vivo assessment of cardiac function with development, WT and KO rats at 3, 6, 9, and 12 months old (8 rats per group) were used. For the in vivo assessment of cardiac function with S107 treatment, WT (8 per group), and KO (9 per group) rats were used. Rats were assessed at day 1 right before S107 treatment and at day 14 right after S107 treatment. Cardiac geometry and function were evaluated under anesthesia using the two‐dimensional guided M‐mode echocardiography (Philips SONOS 5500, Phillips Medical Systems) equipped with a 15–6 MHz linear transducer (Phillips Medical Systems). The detailed procedure of echocardiograph can be referred to previous publication (Ren et al., 2017).

### 2.3 Pressure‐volume loop analysis (PV‐loop)

Rats were anesthetized by intramuscular injection of ketamine hydrochloride (80 mg·kg^−1^ body mass) and xylazine (12 mg·kg^−1^). PE‐50 catheters were surgically placed in the right femoral and jugular veins. The femoral vein catheter was employed for continuous intravenous infusion of a ketamine/xylazine mixture (40/6 mg·ml^−1^, respectively, at a rate of 0.12 ml·h^−1^), which maintained anesthesia throughout the experiment. The jugular vein catheter was for hypertonic saline infusion. The right carotid artery was exposed, and a PV microcatheter (Millar Model 838) was passed through the artery into the left ventricle. The catheter was placed to maximize conductance signal fidelity. The catheter was connected to a Millar Ultra PV device, coupled to an AD Instruments data acquisition system (Model 8SP). Millar Ultra Control Interface software was used to calibrate and receive data from the pressure‐volume catheter, and Lab Chart 8 Pro with PV‐Loop plug‐in was used to store and visualize PV loops, perform saline and cuvette calibrations, and extract data from the recorded loops. After catheter placement in the left ventricle, a 10 min stabilization period ensured consistent baseline PV loops. The inferior vena cava just caudal to the diaphragm was then exposed and briefly occluded with forceps to reduce venous return to the heart. This created PV loops through a range of end‐diastolic volumes. A bolus of hypertonic saline (approximately 40 µl of 30% saline) was then introduced through the jugular catheter, to allow for post‐hoc parallel conductance subtraction. The rat was then heparinized (1,000 USP units in a 0.5 ml bolus of buffer), decapitated, and blood collected for volume cuvette calibration.

### 2.4 Myofilament resting tension measurements

Myofibrils were isolated from 3‐month‐old WT or Rbm20 KO rats (Lin et al., 2020; Woulfe et al., 2019). Left ventricle (LV) sections were skinned in 0.5% Triton‐X in rigor solution (132 mM NaCl, 5 mM KCl, 1 mM MgCl_2_, 10 mM Tris, 5 mM EGTA, pH 7.1) with protease inhibitors (10 µM leupeptin, 5 µM pepstatin, 200 µM PMSF, and 10 µM E64) and 500 µM NaN_3_ and 500 µM DTT at 4℃ overnight. Then skinned tissue was washed in fresh rigor solution and homogenized (Tissue‐Tearor, Thomas Scientific) in relaxing solution (pCa 9.0) containing protease inhibitors. Myofibril suspensions were transferred to a temperature‐controlled chamber (15℃) containing relaxing solution (pCa 9.0; 100 mM Na_2_EGTA; 1 M potassium propionate; 100 mM Na_2_SO_4_; 1 M MOPS; 1 M MgCl_2_; 6.7 mM ATP; and 1 mM creatine phosphate; pH 7.0). Myofibril bundles were mounted between two micro‐tools. The first micro‐tool was connected to a motor that produced rapid length changes (Mad City Labs). The second tool was a calibrated cantilevered force probe (compliance of 11.3 µm/µN). Next, 7–10 myofibrils were stretched incrementally with resting tension recorded at different sarcomere lengths at pCa 9. Sarcomere lengths and myofibril diameters were measured using ImageJ software. Data were collected and analyzed using customized LabView software. The data from all myofibrils of the same animal were grouped into 0.15‐µm intervals of sarcomere length and averaged. The averaged data of all animals from each group were shown as mean ± (or +) SEM values fitted by third‐order polynomials.

### 2.5 Isolation of adult rat cardiomyocyte isolation

Rats at approximately 6 months of age were injected with 60 mg/kg ketamine and 8 mg/kg xylazine (i.p.). Hearts were excised when animals under deep anesthesia. The heart then was cannulated via the aorta. The heart was perfused at 37℃ for 4 min with perfusion buffer (in mM: 113 NaCl, 4.7 KCl, 0.6 Na_2_HPO_4_, 1.2 MgSO_4_, 12 NaHCO_3_, 10 KHCO_3_, 10 HEPES, and 30 taurine, pH 7.4) equilibrated with 5% CO_2_–95% O_2_, followed by perfusion with digestion buffer (perfusion buffer plus 0.45 mg/ml collagenase II [240 U/mg; Worthington Biochemical Corporation], 0.13 mg/ml trypsin, and 25 µM CaCl_2_) for 8–10 min. When the heart was flaccid, digestion was halted and the heart was placed in myocyte stopping buffer 1 (perfusion buffer plus 0.04 ml bovine calf serum [BCS]/ml buffer and 5 µM CaCl_2_). The LV was cut into small pieces, and the rest of the heart was discarded. The small pieces were then triturated several times with a transfer pipette and then filtered through a 100‐µm nylon mesh filter. After this, the cells were gravity pelleted and the supernatant was discarded. Next, 10 ml of myocyte stopping buffer 2 was added (perfusion buffer plus 0.05 ml BCS/ml buffer and 12.5 µM CaCl_2_), and Ca^2+^ was reintroduced to a final concentration of 1.0 mM. At this point. The cardiomyocytes were ready for experiments. This procedure was followed previous publication (Guo et al., 2018).

### 2.6 Cell shortening/relengthening

Rod‐shaped cardiomyocytes with clear edges were selected for treatment of S107 and measurement of mechanical properties with a SoftEdge Myocam system (IonOptix). Cardiomycoytes were treated with 10 µM of freshly prepared S107 and incubated for 2–3 h at room temperature prior to the experiments and cardiomyocytes treated with vehicle were served as control. Myocytes were then loaded on the SoftEdge Myocam system. IonWizard software was used to capture changes in cardiomyocyte length during shortening and relengthening by using the SoftEdge and SarcLen acquisition modules to record cell and sarcomere length. Cardiomyocytes were placed in a C‐Stim Cell Micro Controls superfusion chamber system (IonOptix) on the stage of an inverted microscope (Olympus) and were superfused with the contractile buffer containing (in mM) NaCl 131, KCl 4, MgCl_2_ 1, glucose 10, HEPES 10, and CaCl_2_ 2 (pH7.4). Cardiomyocytes were field stimulated with an acute MyoPacer field stimulator (IonOptix) to electrically pace cellular contractions. The MyoPacer frequency setting for cardiomyocyte contractility measurement was 1 Hz, with a stimulation pulse duration of 3 ms, at the voltage of 15 V. The cardiomyocyte being measured was displayed on the computer monitor via a MyoCam‐S (IonOptix) digital acquisition camera, and the amplitude and velocities of shortening and relengthening were recorded. Cell shortening and relengthening were assessed by using the following indices: peak shortening (PS), the shortest sarcomere length of cardiomyocytes contracted on electrical stimulation, which is indicative of peak ventricular contractility; time‐to‐PS (TPS), the duration of myocyte shortening, which is indicative of contraction duration; time to 50% relengthening (TR_50_), the time to reach 50% relengthening, which represents cardiomyocyte relaxation duration (50% rather than 100% relengthening was used to avoid the noisy signal present at baseline contraction); and maximum velocities of shortening (+dl/dt) and relengthening (−dl/dt), maximum slope (derivative) of the shortening and relengthening phases, which are indicators of maximum velocities of ventricular pressure increase and decrease (Wang et al., 2019).

### 2.7 Intracellular Ca^2+^ transient measurement

Myocytes were loaded with S107 as described above and then Fura‐2 AM (0.5 µM) for 10 min, and fluorescence measurements were recorded with a dual‐excitation fluorescence photomultiplier system (IonOptix). Myocytes were placed on an Olympus IX‐70 inverted microscope stage and imaged through a Fluor x40 objective. Myocytes were exposed to light emitted by a 75‐W lamp and passed through either a 360‐ or a 380‐nm filter while being stimulated to contract at 0.5 Hz. Fluorescence emissions were detected between 480 and 520 nm by a photomultiplier tube after first illuminating the myocytes at 360 nm for 0.5 s and then at 380 nm for the duration of the recording protocol (333 Hz sampling rate). The excitation scan at 360 nm was repeated at the end of the protocol and qualitative changes in intracellular Ca^2+^ levels were inferred from the ratio of fura‐2 fluorescence intensity (FFI) at the two wavelengths (360/380). Fluorescence decay time was measured as an indication of the intracellular Ca^2+^ clearing rate. Single exponential curve fit programs were used to derive the intracellular Ca^2+^ decay constant (Guo et al., 2018; Ren et al., 2017; Wang et al., 2019).

### 2.8 Titin gel electrophoresis

Titin isoforms were resolved as previously described using a vertical sodium dodecyl sulfate (SDS)‐1% agarose gel electrophoresis (VAGE) system (Warren et al., 2003, 2004; Zhu & Guo, 2017). The frozen tissue samples from 3‐, 6‐, and 9‐month‐old rat hearts were dissolved in urea‐thiourea‐SDS‐dithiothreitol sample buffer using a small Dounce homogenizer or a Mini BeadBeater (Biospec Products) and were heated for 10 min at ~65℃. Silver stained agarose gels were dried between sheets of mylar and dried gels were scanned. Full details have been discussed in a previous published report (Warren et al., 2003, 2004; Zhu & Guo, 2017).

### 2.9 Western blotting and RT‐PCR

Protein samples were prepared from heart tissues of 6‐month‐old WT and KO rats. Proteins were then separated by SDS‐PAGE gel and transferred onto a PVDF membrane. The membrane was probed with antibodies against RyR2_pSer2808 (ThermoFisher Scientific, Cat#PA5‐105712), RyR2 (Abcam, Cat#ab219798). Mouse anti‐rabbit IgG‐conjugated with horseradish peroxidase (Fisher Scientific) was served as the secondary antibody. GAPDH (Cell Signaling Technology, Cat#2118) was served as the protein loading control. The detailed procedure was described previously (Guo et al., 2018). RNA samples were prepared from the same tissues as in the protein samples. Total RNA was extracted in 1 ml TRIzol (Invitrogen), following manufacturer instructions. RNA was precipitated from the TRIzol reagent with isopropanol and resuspended at 0.5–1.0 mg/ml using RNA secure solution (Ambion). RNA samples were treated with DNaseI (DNA‐free; Ambion). After extraction, all samples were stored at −80 degree. RNA concentration was measured with a NanoDrop ND 1000 spectrometer (NanoDrop Technologies), and RNA integrity was assessed on an Agilent 2100 Bioanalyzer (Agilent Technologies). RNA was further purified by the RNeasy mini kit (Qiagen). For reverse transcription, 60 ng of RNA was mixed with 5 µM random hexamers, 1 mM each dNTP, 7.5 mM MgCl2, 40 U RNasin (Promega), 1X PCR buffer II (Applied Biosystems), and 250 U of SuperScript II reverse transcriptase (ThermoFisher Scientific). The reaction mixture was incubated at 25℃ for 10 min, 48℃ for 45 min, and 95℃ for 5 min, then cooled down to 4℃. Two volumes of ethanol were added to the cDNA and evenly mixed, and then stored at −20℃ for 30 min, centrifuging for 15 min at 13,000 rpm, washed with 75% ethanol and dried. The cDNA was resuspended in distilled water and used as a template. RT‐PCR was carried out with primers spanning Ryr2 exon 76–77 (Forward: 5′‐CAGCCTTAACAGAGAAATGC‐3′ and Reverse: 5′‐CTCTCAAAGGCATTCAGGTC‐3′).

### 2.10 Statistical analysis

GraphPad prism software was used for statistical analysis. Results were expressed as means ± SEM. Statistical significance between groups was determined using one‐way ANOVA with a Tukey's posttest, or a two‐tailed *t*‐test for comparison of two groups. The significance level was *p < .05, **p < .01, ***p < .001.

## 3 RESULTS

### 3.1 Depletion of Rbm20 results in larger titin isoform expression, lower resting myofilament tension, and diastolic stiffness in rats

**FIGURE 1:**
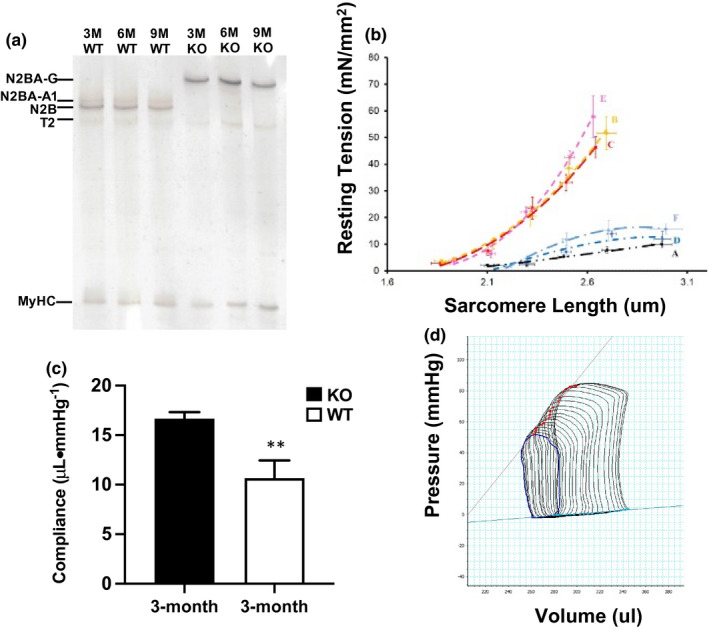
Titin size switching and resting stiffness with in vitro and in vivo measurements. (a) Detection of titin size switching in WT and KO rat heart with ageing using gel electrophoresis; (b) resting tension measurements with in vitro isolated individual myofibrils in WT group (three biological repeats C, B, and E) and in KO group (three biological repeats A, D, and F); (c) diastolic stiffness measured with PV‐loop in 3 months old WT and KO rats; (d) representative example of PV loops of an inferior vena cava (IVC) occlusion experiment; the end‐diastolic PV relationship (EDPVR) is shown as a blue line. KO, RBM20 knockout; M, month; MyHC, myosin heavy chain; N2B, smaller titin isoform; N2BA‐A1 and G, larger titin isoforms; T2, degraded titin; WT, wild type; Mean ± SEM (*n* = 3 and 6), *p < .05, **p < .01

RBM20 predominantly regulates pre‐mRNA splicing of gene TTN, encoding a largest sarcomeric protein titin in muscle tissues (Guo et al., 2012, 2013; Li et al., 2013). In the heart of 3, 6, and 9‐month‐old WT rats, a smaller titin isoform N2B is predominately expressed, while a single larger titin isoform N2BA‐G is expressed in the heart of Rbm20 KO rats (Figure 1a). The ratio of the titin isoforms is a major indicator for resting tension of the myofilament (Guo et al., 2010; Guo & Sun, 2018; Tharp et al., 2020). To assess if changes in the ratio of titin isoforms presented in Rbm20 KO rats lead to decreased resting tension, myofibrils were isolated from the left ventricle of 3‐month‐old WT and KO rats. The myofibrils from the KO hearts produced lower resting tension compared to WT myofibrils (Figure 1b). Furthermore, reduced resting tension in cardiomyocytes may also contribute to overall myocardial stiffness. To this end, we performed pressure‐volume loop (PV‐Loop) study with 3‐month‐old rats. We observed that diastolic stiffness is significantly decreased in KO rats at the age of 3 months (Figure 1c). Figure 1d is a representative example of PV loops of an inferior vena cava (IVC) occlusion experiment and the end‐diastolic PV relationship (EDPVR) was shown in blue line (Figure 1d). Compliance is the reciprocal of EDPVR slope.

### 3.2 Rbm20 KO leads to overt ventricular dilation and reduced contractile function by 12 months of age

**FIGURE 2:**
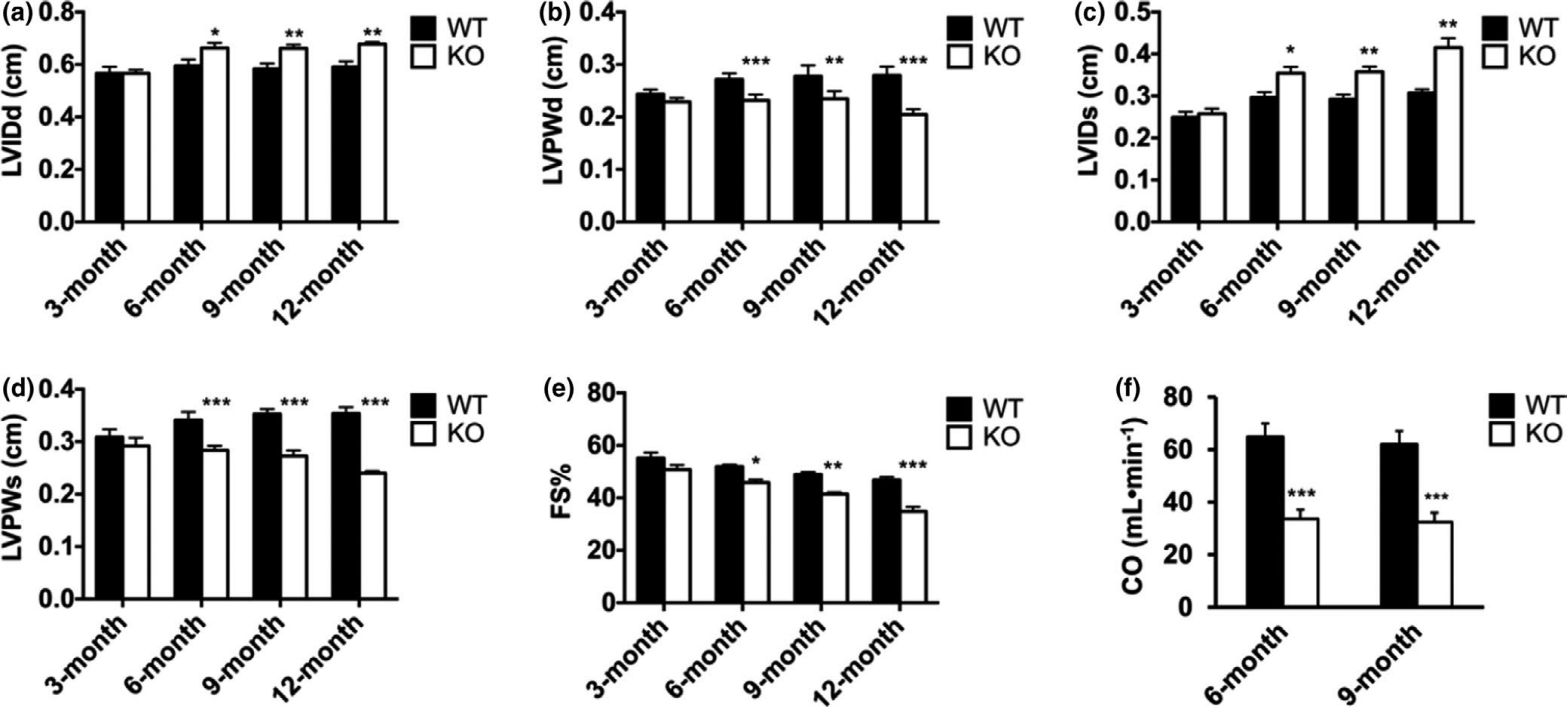
In vivo assessment of cardiac function. (a) and (c) Increase in left ventricle (LV) internal dimension in both diastole and systole in KO at the age of 3 (*n* = 8 and 9), 6 (*n* = 7 and 12), 9 (*n* = 7 and 10), and 12 months (*n* = 6 and 6); (b) and (d) decrease in LV posterior wall dimension in both diastole and systole in KO rat at the age of 3, 6, 9, and 12 months; (e) fractional shortening value decreases in KO rat at the age of 3, 6, 9, and 12 months; (f) cardiac output decreases in KO at 6 and 9 months determined by PV‐Loop analysis. CO, cardiac output; FS, fractional shortening; KO, RBM20 knockout; LVIDd, left ventricular end‐diastolic internal dimension; LVIDs, left ventricular end systolic internal dimension; LVPWd, left ventricular end‐diastolic posterior wall dimension; LVPWs, left ventricular end systolic posterior wall dimension; WT, wild type. Mean ± SEM, *p < .05, **p < .01, ***p < .001

Animals lacking RBM20 are predisposed to developing dilated cardiomyopathy and heart failure (Guo et al., 2012; van den Hoogenhof et al., 2018; Khan et al., 2016). However, it is still unclear at what developmental stage Rbm20 depletion causes overt phenotypical changes of cardiac morphology and geometry. We assessed cardiac function in Rbm20 KO rats at ages of 3, 6, 9, and 12 months. The results demonstrated that there were no any significant differences of left ventricular end‐diastolic internal dimension (LVIDd), left ventricular end‐diastolic posterior wall dimension (LVPWd), left ventricular end systolic internal dimension (LVIDs), left ventricular end systolic posterior wall dimension (LVPWs) and fractional shortening (FS) between WT and KO rats at age of 3 months (Figure 2a–e). However, significant dilated ventricular chamber, thinner posterior wall and reduced fractional shortening at ages of 6, 9, and 12 months (Figure 2a–e, Table S1). PV‐loop analysis at age of 6 and 9 months showed decreased cardiac output in Rbm20 KO rats when compared to WT rats (Figure 2f). These data show enlarged LV chamber size and thinner LV wall thickness in both diastole and systole phases in KO rat at and after approximately 6 months of age and beyond.

### 3.3 Loss of Rbm20 results in impaired contractile properties that was restored by S107 treatment in adult rat cardiomyocytes

**FIGURE 3:**
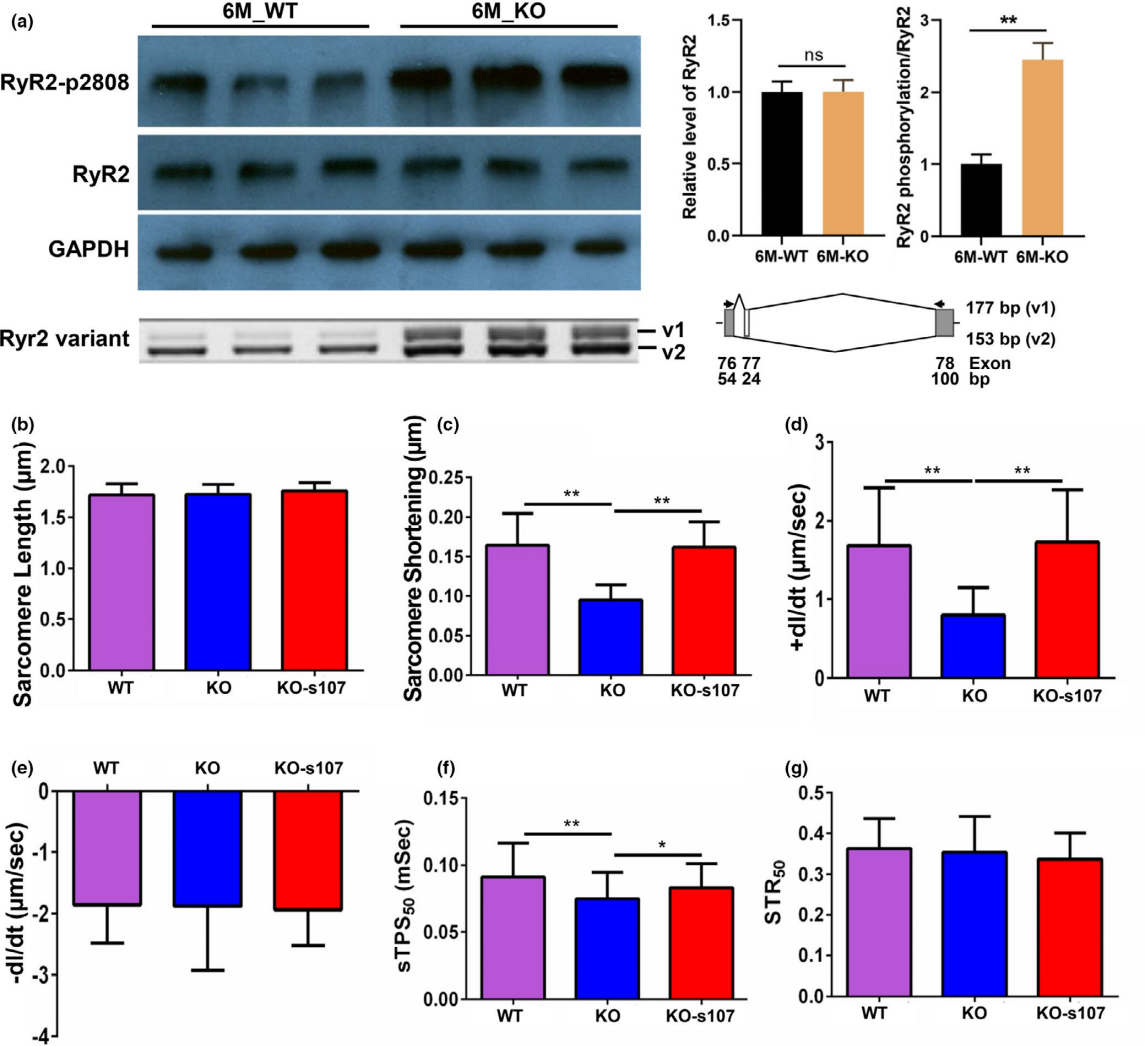
Ryr2 variant and cardiomyocyte contractility measurements in WT and KO cardiomyocytes treated with and without S107. (a) RyR2 expression, phosphorylation and splicing variant; (b) resting sarcomere length treated with S107; (c) peak shortening treated with S107; (d) maximum velocity of shortening treated with S107; (e) maximum velocity of relengthening treated with S107; (f) time to 50% peak shortening treated with S107; (g) time to 50% relengthening treated with S107; KO, knockout; sTPS_50_, time to 50% peak shortening; sTR_50_, time to 50% relengthening; WT, wild type. Mean ± SEM (*n* = 100 cells from three biological replicates), *p < .05, **p < .01

Previous reports indicated that mutations in Rbm20 clinically cause severe form of dilated cardiomyopathy (DCM) (Brauch et al., 2009; Li et al., 2010; Refaat et al., 2012). Depletion and mutations of Rbm20 in rodent and pig models have also shown severe DCM and arrhythmias (Guo et al., 2012; van den Hoogenhof et al., 2018; Ihara et al., 2020; Khan et al., 2016; Schneider et al., 2020). Splicing events regulated by RBM20 involve in Ca^2+^ handling genes such as RyR2 that in turn impair the contractility of cardiomyocytes in rodents with loss of Rbm20 through pro‐arrhythmic Ca^2+^ releases from the sarcoplasmic reticulum (SR) (Guo et al., 2018; van den Hoogenhof et al., 2018). Western blotting showed that total RyR2 was not changed, but RyR2_pSer2808 were significantly increased in Rbm20 KO by comparing to WT rats (Figure 3a). Furthermore, RT‐PCR indicated that the larger transcript variant of Ryr2 was increased in Rbm20 KO heart tissues when compared to WT (Figure 3a). Increased RyR2_pSer2808 destabilizes RyR2‐FKBP12.6 (also known as calstabin 2) complex, resulting in leaky RyR2 (Shan, Betzenhauser, et al., 2010). The altered splicing variant may also interrupt the RyR multi‐protein complex and thus results in impaired SR Ca^2+^ release. To validate this, S107, a stabilizer of RyR multi‐protein complex was used to test whether S107 prevents RBM20‐induced SR Ca^2+^ release and improve cardiomyocyte contractile properties, we isolated adult rat cardiomyocytes and measured the cardiomyocyte contractility using IonOptix contractility measurement system. The results revealed that loss of Rbm20 does not impact sarcomere length and addition of S107 has no effect on sarcomere length (Figure 3b). Cardiomyocyte contractility showed that loss of Rbm20 decreased peak shorting (PS) and maximal velocity of sarcomere shortening (+dL/dt) associated with reduced time to 50% peak shortening (sTPS_50_) in Rbm20 KO cardiomyocytes compared with those from WT cardiomyocytes (Figure 3c,d,f). RyR2 stabilizer S107 treatment restored all of these impaired contractile properties in KO cardiomyocytes to the level of WT cardiomyocytes (Figure 3c,d,f). However, maximal velocity of sarcomere relengthening (−dL/dt) and time to 50% relengthening (sTR_50_) were not suppressed by loss of Rbm20. Also, S107 had no impact on −dL/dt and sTR_50_ (Figure 3e,g). These data demonstrate decreased PS, along with slower speed of shortening and suppressed sTPS_50_ is likely due to Ca^2+^ leaking caused by loss of Rbm20. On the other hand, the speed of relengthening and sTR50 was unaffected by loss of Rbm20, meaning Ca^2+^ uptake into the sarcoplasmic reticulum was not impaired by loss of Rbm20.

### 3.4 Rbm20 depletion impairs intracellular Ca^2+^ homeostasis and S107 restores resting Ca^2+^ level in adult rat cardiomyocytes

**FIGURE 4:**
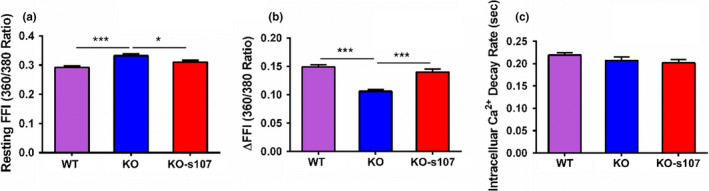
Intracellular calcium transient measurements. (a) Resting fura‐2 fluorescence intensity (FFI) with S107 treatment; (b) electrically stimulated rise in fura‐2 fluorescence intensity (∆FFI) with S107 treatment; (c) intracellular calcium decay rate (single exponential) with S107 treatment. KO, knockout; WT, wild type. Mean ± SEM (*n* = 100 cells from three biological replicates), *p < .05, ***p < .001

We further explored whether impaired contractile properties are associated with intracellular Ca^2+^ release in response to loss of Rbm20. Intracellular Ca^2+^ levels were assessed with the fura‐2 fluorescence technique and measured with IonOptix contractility system. Rbm20 depletion significantly elevated the baseline of fura‐2 fluorescence intensity (FFI) and suppressed electrically stimulated rise in fura‐2 fluorescence intensity (∆FFI), while Rbm20 depletion did not impact intracellular Ca^2+^ decay rate when compared to WT cardiomyocytes (Figure 4a–c). Addition of S107 to the Rbm20 KO cardiomyocytes abolished the elevated baseline FFI and ameliorate the electrically stimulated rise in ∆FFI without affecting intracellular Ca^2+^ decay rate (Figure 4a–c). These data show that Rbm20 depletion impairs RyR2‐gated Ca^2+^ release channel, leading to increased intracellular Ca^2+^ overload in the resting cardiomyocytes. However, the RyR2 stabilizer S107 inhibited Ca^2+^ leaking and restored the resting intracellular Ca^2+^ level, and thus improve the electrically stimulated rise in ∆FFI. On the other hand, our data further show that Ca^2+^ uptake into the sarcoplasmic reticulum is not impaired by Rbm20 depletion.

### 3.5 Treatment with S107 in Rbm20 knockout rats did not improve cardiac function

**FIGURE 5:**
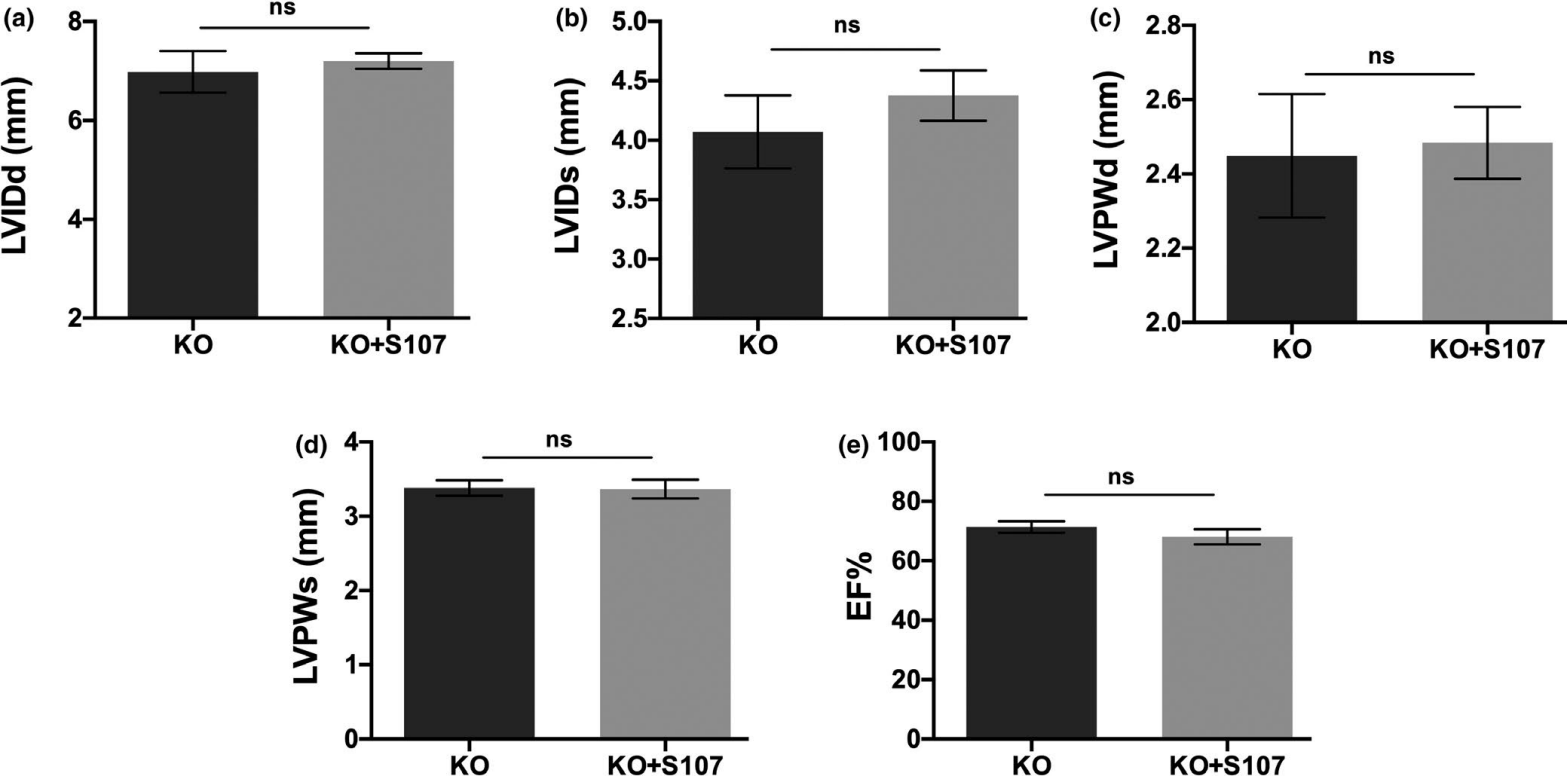
In vivo assessment of cardiac function with S107 treatment. (a) and (b) Left ventricle (LV) internal dimension in both diastole and systole in KO rats with/without S107 treatment; (c) and (d) LV posterior wall dimension in both diastole and systole in KO rats treated with/without S107; (e) ejection fraction in KO rats treated with/without S107. EF, ejection fraction; KO, RBM20 knockout; LVIDd, left ventricular end‐diastolic internal dimension; LVIDs, left ventricular end systolic internal dimension; LVPWd, left ventricular end‐diastolic posterior wall dimension; LVPWs, left ventricular end systolic posterior wall dimension. Mean ± SEM (*n* = 9 and 6), ns, not significant.

Our data shown in Figure 4 confirmed that impaired contractile properties observed in Figure 3 were associated with elevated intracellular Ca^2+^ release through the in vitro measurements of contractility properties and Ca^2+^ transient. Also, the RyR2 stabilizer S107 restored the elevated intracellular Ca^2+^ level to normal and ameliorated Rbm20 depletion‐induced abnormal contractility. These findings lead us to further test whether S107 can improve cardiac function in Rbm20 KO rats. Rbm20 KO rats at 1‐year‐old were treated with S107 in drinking water for 2 weeks. Cardiac function was assessed with echocardiograph at day 1 before treatment. After 2 weeks treatment, in vivo cardiac function was assessed again with echocardiograph. Before treatment, enlarged LV chamber size, thinner LV wall thickness and ejection fraction (EF) in both diastole and systole phases in Rbm20 KO rats were significantly lower than that of WT rats at the age of 12 months (Figure 2). Unexpectedly, after treatment, we did not observe significant improvement of enlarged LV chamber size, thinner LV wall thickness and EF in Rbm20 KO rats treated with S107 by comparing to the same animals before treatment (Figure 5a–e).

## 4 DISCUSSION

Our findings revealed that deletion mutation with loss of Rbm20 in rat results in increased expression of the larger titin isoform and reduced myofibril stiffness at an early age. However, overt phenotype of enlarged left ventricular chamber, thinner ventricular walls and reduced cardiac output appears at older age. Loss of RBM20 increases resting intracellular Ca^2+^ levels in in vitro isolated cardiomyocytes through impairing SR Ca^2+^ release channel gated by RyR2. The potential mechanisms could be associated with increased RyR2 phosphorylation and the change of RyR2 splicing variant regulated by RBM20 (Guo et al., 2018; Maatz et al., 2014). The RyR2 stabilizer S107 restores the resting intracellular Ca^2+^ level and thus improves the contractile properties in Rbm20 KO adult rat cardiomyocytes. Unexpectedly, our results showed that S107 treatment in Rbm20 KO rats does not improve cardiac function significantly.

Previous and recent studies with Rbm20 KO in both rat and mice and Rbm20 truncation deletion in mice manifest very different phenotype, respectively (Guo et al., 2012; Khan et al., 2016; Methawasin et al., 2014). In our rat model, 13 of 14 exons were depleted but exon 1, leading to loss‐of‐function of RBM20. Our rat model increases the expression of larger titin isoform and exhibits DCM and arrythmias (Guo et al., 2012). Later, two mouse models were generated by two groups, respectively. In Dr. Granzier's group, they generated a mouse model with RNA recognition motif (RRM) domain (exons 6 and 7) deleted. These mice still produce a truncated RBM20 (*Rbm20^∆RRM^*). Although these mice express exclusively larger titin isoform which is consistent with our Rbm20 KO rats, they do not show similar phenotype as observed in our KO rats with even older age of these mice (Methawasin et al., 2014). In another group, they generated another mouse line by removing exons 4 and 5 that results in no RBM20 expression in mice (Khan et al., 2016). This Rbm20 KO mouse model exactly phenocopies our KO rat model by which the Rbm20 KO mice increase the expression of larger titin isoform, develop DCM and exhibit arrhythmias (van den Hoogenhof et al., 2018; Khan et al., 2016). Although both Rbm20 KO rats and mice develop overt DCM phenotype, it is unclear at what developmental age the rats and mice display apparent DCM phenotype. In this study, by using our Rbm20 KO rat model, we found that these rats display the overt DCM phenotype at age of about 6 months and later age (Figure 2).

The in vivo assessment of cardiac function with development in Rbm20 KO rats provided us a guidance for at what age the rats need be used for S107 treatment. The rycal S107, a RyR‐Calstabin interaction stabilizer, is known to inhibit SR Ca^2+^ leaking via RyR2 by inhibiting dissociation of Calstabin 2 (also known as FKBP12.6) from RyR2 (Shan, Betzenhauser, et al., 2010). Calstabin 2 selectively binds to RyR2 and stabilizes the closed state of RyR2, thus, preventing resting Ca^2+^ release (Wehrens et al., 2003, 2004; Yuan et al., 2016). In this study, we chose 6‐month‐old rats to isolate cardiomyocytes and treated cardiomyocytes with S107. Our data demonstrated that S107 can bring back the resting intracellular Ca^2+^ level to normal in Rbm20 KO cardiomyocytes, suggesting dissociation between RyR2 and Calstabin 2 occurs. A handful of studies have shown that PKA and CamkIId phosphorylation, oxidation, and nitrosylation of RyR2 dissociates Calstabin 2 from the RyR2 channel, leading to resting Ca^2+^ leaking (Chelu et al., 2009; Li, Wang, et al., 2012; Shan, Betzenhauser, et al., 2010; Shan, Kushnir, et al., 2010; Wehrens et al., 2005, 2006). One of the possible reasons for dissociation between RyR2 and Calstabin 2 in Rbm20 KO cardiomyocytes could be increased RyR2 phosphorylation level caused by PKA or CamkIId. Interestingly, the splicing variants of CamkIId are also regulated by RBM20 (Guo et al., 2012; Maatz et al., 2014; Methawasin et al., 2014), which could be the cause of increased RyR2 phosphorylation. Whether camkIId gene splicing modulates RyR2 phosphorylation need to be further studied in the future. Another possible reason is that RBM20 can directly regulate alternative splicing of RyR2, leading to inclusion of 24 bp from RyR2 mRNA and a larger isoform expression. Whether RBM20‐mediated CamkIId gene splicing impacts on RyR2 phosphorylation and/or whether larger RyR2 isoform resulting from RBM20‐regulated alternative splicing facilitates unstable interaction between RyR2 and Calstabin 2 remain unknown and need to be further studied.

On the other hand, we further investigated whether restored resting intracellular Ca^2+^ concentration and improved contractile properties in the in vitro isolated cardiomyocytes by S107 can be also applied to live animals to ameliorate in vivo cardiac function. Unexpectedly, we did not observe improvement of cardiac function in Rbm20 KO rats treated with S107. However, this is not surprising because of the multiple causes for Rbm20 depletion‐induced cardiac dysfunction as well as experimental limitations. As discussed above, both titin size switching and impaired Ca^2+^ handling are primary contributors to Rbm20 depletion‐induced cardiac dysfunction. In addition, RBM20 also regulate the splicing of 30 other cardiac genes, most of which have been previously associated with DCM and heart failure (Guo et al., 2012; Linke & Bücker, 2012). Some of these genes encode proteins that are involved in ion transport and sarcomere structure and function such as myosin heavy chain and cyber (Guo et al., 2012; Linke & Bücker, 2012; Maatz et al., 2014). Most recently, Rbm20 mutation knock‐in pigs and mice show relocation of RBM20 and RNA granules formation (Ihara et al., 2020; Schneider et al., 2020). Therefore, it could be possible that targeting only Ca^2+^ signaling may not significantly prevent the development of DCM or reverse the cardiac dysfunction caused by the combination of multiple conditions. In addition, we treated the animals at 1‐year‐old age which could be past the point of reverse remodeling of the heart since the KO animals develop overt DCM phenotype at about 6‐month‐old. Therefore, treatment at early age such as at or before the age of 6 months could prevent the development of DCM. Further, cardiomyocytes isolated from 12‐month‐old rats treated with S107 could be done in the future to indicate past the point of reverse remodeling of the heart. Other possibilities from experimental limitations could be the duration of treatment and dosage of S107. Previous report showed that 10 weeks of treatment with 20 mg/kg/day of S107 in RyR2 mutation knock‐in mice with osmotic pump can significantly preserve cardiac function (Shan, Betzenhauser, et al., 2010). We used the same dosage but in drinking water and we only treated rats for 2 weeks which may not be sufficient to elicit pharmacological effect. Future study needs higher and more controlled delivery methods as described previously (Shan, Betzenhauser, et al., 2010) to obtain sufficient pharmacological effect. Lastly, whether sex may also play a role in the effective treatment of S107 to prevent DCM development is unclear. Hence, our in vivo data may not offer conclusive answers to whether or not S107 can ameliorate the cardiac dysfunction affected by Rbm20 deletion in rats.

In summary, our findings suggest that even though titin size switching occurs and myocardial stiffness is changed at younger age of Rbm20 KO rats, the overt phenotype of heart failure exhibits at older age. It appears that alterations in myocardial stiffness as well as altered Ca^2+^ handling drives the development of DCM in older rats. However, it remains unknown whether correcting Ca^2+^ handling at a younger age in this rat model may delay or lessen the severity of DCM disease developed. The RyR2 stabilizer S107 restores resting intracellular Ca^2+^ level and improve contractile properties in the in vitro studies. However, in this study, we did not observe cardiac function improvement in the in vivo studies with S107 treatment. Whether S107 can improve cardiac function, alter the spontaneous calcium spark, and affect the level and localization of RyR2 in Rbm20 KO rats deserves further investigation.

## CONFLICT OF INTEREST

None.

## AUTHOR CONTRIBUTIONS

W.G., H.G.C., J.R., and T.A.M. designed the experiments; Z.C., H.G.C., Y.Z., A.S.W., L.Y.H., and K.C.W. performed the experiments; W.G., H.G.C., J.R., T.A.M., Z.C., Y.Z., Z.Y., W.C., Y.H.L., and K.C.W. analyzed and interpreted the data; W.G. wrote the manuscript; H.G.C., J.R., T.A.M., and K.C.W. revised the manuscript.

## Supporting information



Table S1
